# Low‐fat dairy consumption and the risk of lung cancer: A large prospective cohort study

**DOI:** 10.1002/cam4.6249

**Published:** 2023-06-16

**Authors:** Zhiyong Zhu, Linglong Peng, He Zhou, Haitao Gu, Yunhao Tang, Zhihang Zhou, Ling Xiang, Yaxu Wang

**Affiliations:** ^1^ Department of Gastrointestinal Surgery The Second Affiliated Hospital of Chongqing Medical University Chongqing China; ^2^ Laboratory of Cancer Biology Department of Oncology University of Oxford Oxford UK; ^3^ The Second Department of Gastrointestinal Surgery Affiliated Hospital of North Sichuan Medical College Nanchong China; ^4^ Department of Gastroenterology The Second Affiliated Hospital of Chongqing Medical University Chongqing China; ^5^ Department of Clinical Nutrition The Second Affiliated Hospital of Chongqing Medical University Chongqing China

**Keywords:** Cox regression analysis, low‐fat dairy, lung cancer, PLCO trial

## Abstract

**Background:**

Despite the possible contribution of dairy products to the development or prevention of cancers, there is a lack of epidemiological evidence linking low‐fat dairy consumption to the risk of developing lung cancer. This research was conducted to fill this knowledge gap.

**Methods:**

The data for this research were collected from the Prostate, Lung, Colorectal, and Ovarian (PLCO) Cancer Screening Trial. The Cox proportional risk model was employed to evaluate the link between low‐fat dairy consumption and the risk of developing lung cancer. Hazard ratios (HRs) and 95% confidence intervals (CIs) were measured in both unadjusted and adjusted models. A series of predefined subgroup analyses were performed to identify potential effect modifiers, and several sensitivity analyses were conducted to assess the stability of the findings.

**Results:**

The study included data from 98,459 individuals. During a total of 869,807.9 follow‐up person‐years, 1642 cases of lung cancer were observed, with an incidence of 0.189 cases for every 100 person‐years. In the fully adjusted model, participants in the highest quartile of low‐fat dairy consumption had a significantly decreased risk of lung cancer compared to the ones in the lowest quartile (HR_quartile 4 vs. 1_: 0.769, 95% CI: 0.664, 0.891, *p*_trend_ = 0.005). The restricted cubic spline plot revealed an inverse nonlinear dose–response relationship between low‐fat dairy consumption and lung cancer risk (*p*_nonlinearity_ = 0.008). Subgroup analyses demonstrated that the inverse association was stronger among participants with higher daily caloric intake (*p*_interaction_ = 0.031). Various sensitivity analyses produced consistent results.

**Conclusion:**

Consuming more low‐fat dairy products is significantly linked to a reduced risk of developing lung cancer, indicating that an appropriate increase in the use of low‐fat dairy products may help prevent lung cancer.

## 1 INTRODUCTION

With about 2.2 million new cases diagnosed in 2020, lung cancer ranks as the second most prevalent cancer globally, representing 11.4% of all cancer cases.^1^ Despite the advances in therapeutic strategies, it remains the primary cause of tumor‐related deaths and a major global health burden.^2^, ^3^ While smoking is a well‐established risk factor, growing epidemiological evidence suggests that certain dietary factors, such as fruits, dietary fiber, vegetables, and red and processed meats, may also influence the incidence rate of lung cancer.^4^, ^5^, ^6^ Identifying additional nutritional factors associated with lung cancer may aid its prevention.

The link between dairy intake and various types of carcinoma has been investigated, particularly prostate and breast cancers.^7^, ^8^ However, there has been limited and inconclusive research on the relationship between dairy consumption and lung cancer. For instance, a prospective study in 2020 revealed that eating yogurt may lower lung cancer risk.^9^ In contrast, another investigation discovered no connection between drinking milk and the risk of developing the disease.^10^ Previous evidence suggests that the relationship between dairy products and cancers may vary depending on the fat content and types of dairy products consumed.^11^, ^12^ Therefore, it could be speculated that the inconsistent findings for lung cancer may be due to these differences. Filtering full‐fat dairy products to remove the majority of the saturated fatty acids while preserving the unsaturated fatty acids results in low‐fat dairy products like low‐fat cheese, low‐fat cream, low‐fat or skim milk, and yogurt.^13^ Therefore, the fatty acid contents of low‐fat dairy products and other dairy products are significantly different. In recent years, low‐fat dairy products have replaced their whole‐fat alternatives in dietary guidelines due to their potential health benefits.^14^ The correlation between consuming low‐fat dairy products and the risk of developing lung cancer is still vague. This research investigated this relationship using prospective data from a large US population.

## 2 MATERIALS AND METHODS

### 2.1 Study population and design

The study population was determined from the Prostate, Lung, Colorectal, and Ovarian (PLCO) Cancer Screening Trial, a large, multicenter, prospective study sponsored by the United States.^15^ This investigation aimed to examine whether specific predefined screening tests (e.g., chest radiograph, flexible sigmoidoscopy, etc.) could improve the prognosis of individuals suffering from PLCO cancers. Ten screening centers across the United States recruited and screened participants between November 1993 and September 2001. Eligible candidates were aged 55–74 years old and were invited to participate in the research. The following were the exclusion criteria: (i) individuals who had a history of PLCO cancers; (ii) participants who had treatment for any cancer except basal or squamous cell skin cancer; (iii) individuals who underwent surgery to remove their entire prostate, the entire colon or one lung; (iv) individuals who recently received any screening examination for prostate or colorectal cancer; (v) individuals enrolled in another cancer screening or prevention trial; and (vi) men with a history of recent finasteride use. Ultimately, 154,887 eligible participants were enrolled in the PLCO trial. Each participant provided written consent before entry into the trial. Participants were randomized in equal proportions into control or intervention arms. Participants in the intervention group underwent routinely scheduled PLCO cancer screening examinations, whereas regular care was provided to the individuals in the control group. The baseline questionnaire (BQ) was used to gather baseline data, such as age, gender, race/ethnic, disease history, and medication history. The diet history questionnaire (DHQ) was used to capture dietary information in the past year with self‐reported data, including frequency of certain foods, nutrient intake, etc. The DHQ is a well‐designed dietary assessment tool, and its scientific validity has been confirmed in previous studies.^16^ In both arms, 77% of individuals finished the DHQ. Raw survey results were transformed into variables that were appropriate for analysis in terms of gram intake for foods, frequency of meals, etc. For example, grams of a particular food consumed daily were calculated based on the coded responses to the food frequency and serving size questions by DietCalc.^17^ Each participant was followed up until an outcome event occurred or the follow‐up endpoint (December 31, 2009) was met. Moreover, lung cancer diagnosis, death, or loss to follow‐up were all defined as outcome events, depending on which one occurred first.

**FIGURE 1:**
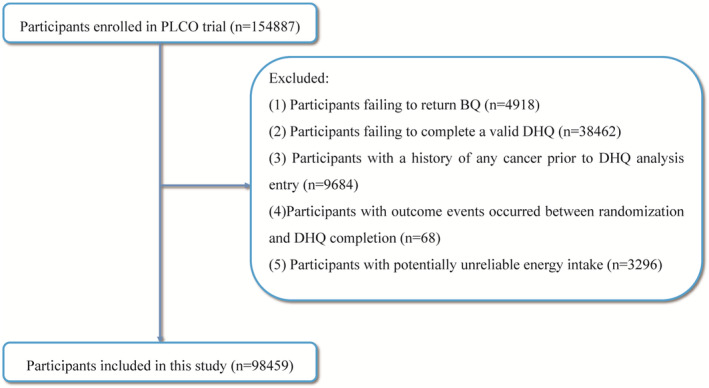
The flow chart of identifying subjects included in our study. BQ, baseline questionnaire; DHQ, diet history questionnaire; PLCO, Prostate, Lung, Colorectal, and Ovarian.

In this research, the following participants were excluded for further analysis: (i) participants failing to return BQ (*n* = 4918); (ii) individuals failing to complete a valid DHQ (*n* = 38,462); (iii) individuals who had a history of any cancer before completing the DHQ (*n* = 9684); (iv) individuals with outcome events occurring prior to DHQ completion (*n* = 68); and (v) participants with extreme daily caloric intake (*n* = 3296), where the extreme daily caloric intake was defined as daily caloric intake >4200 or <800 kcal for males and >3500 or <600 kcal for females.^18^ Figure 1 shows the specific study population inclusion process in this study. This research was carried out with the approval of the National Cancer Institute (NCI, project number: PLCO‐1150).

### 2.2 Assessment of low‐fat dairy intake as well as covariates

This study adopted the definition of “low‐fat dairy products” as per the existing literature and American dietary habits.^19^, ^20^ This encompasses a range of foods, including cottage cheese, low‐fat cream, low‐fat milk, skim milk, and yogurt. Daily consumption of these foods was obtained from the DHQ. Both the total consumption of low‐fat dairy products as well as individual consumption of each component as exposures were evaluated.

For building the adjusted models and subgroup analyses, the following covariates were considered. Data on gender, race/ethnic, marital status, education level, smoking status, trial arm, and family history of lung cancer were obtained from the responses to the BQ, whereas those for age, drinking status, daily caloric and fiber intake, and consuming fruits, vegetables, and red and processed meats were obtained from the responses to the DHQ. Body mass index (BMI) was calculated as weight (kg)/height squared (m^2^).

### 2.3 Ascertainment of lung cancer cases

Lung cancer cases were identified through an annual follow‐up survey administered to the participants via mail. The survey included questions about cancer diagnosis, including the type, timing, and location of the malignancy. Candidates were contacted again by phone or email if they did not reply to the follow‐up form. Furthermore, abnormal suspicious results of chest X‐ray screening, death certificate, lung cancer judgment by local Death Review Committee based on other indicators, and family reports were used as additional materials by the PLCO trial team to identify lung cancer cases and learn more details about this cancer. To ensure that confirmed lung cancer cases were authentic, if single‐source reference such as a death certificate indicated lung cancer, this participant was still not identified as a lung cancer case, which means that other materials mentioned above were necessary to confirm lung cancer diagnosis.

### 2.4 Statistical analysis

This study encountered missing data for several covariates. Among them, BMI, a continuous variable, had the largest proportion of missing data at 1.31%. Other covariates, including a family history of lung cancer, race/ethnic, education level, smoking, and marital status, were all categorical variables and had missing values for >1% of participants. For continuous and categorical variables, missing data were imputed using the median and modal values, respectively.^21^ Table S1 demonstrates the distribution of factors with missing data both before and after imputation. The complete data set, following imputation, was utilized in subsequent analyses.

According to how much low‐fat dairy they consumed each day, participants were split into quartiles, with higher quartiles indicating higher consumption levels. Follow‐up person‐years were separately calculated for each quartile. The incidence rate of lung cancer was determined by dividing the number of cases by person‐years and multiplying by 100 to obtain the rate per 100 person‐years. The Cox proportional risk model was used to determine the link between intake of low‐fat dairy products and the risk of developing lung cancer, with follow‐up time as the timeline. The lowest quartile of participants served as the reference group, and for the other quartiles, hazard ratios (HRs) and 95% confidence intervals (CIs) were measured. Multivariate analyses were performed to control for possible confounders. Demographic data like age (years, continuous), gender (male, female), and race/ethnic (white, non‐white) were taken into account when adjusting Model 1. Additionally, Model 2 was modified for marital status (married or living as married, others), level of education (college below, college graduate or postgraduate), drinking (no, yes) and smoking habits (never smoker, current or former smoker), trial arm (intervention, control), family history of lung cancer (no, yes or possible), daily caloric (kcal, continuous) and fiber (g/day, continuous) intake, and consuming fruits (g/day, continuous), vegetables (g/day, continuous), red and processed meats (g/day, continuous). It is important to note that the adjusted covariates were selected based on existing literature rather than subjective preferences.^22^ Low‐fat dairy intake was assigned as the median for all participants within each quartile to determine if there is a linear trend between consuming low‐fat dairy products and the risk of developing lung cancer. *p*‐value for the trend tests were then calculated separately for the unadjusted and adjusted models. Additionally, the link between each component and the risk of developing lung cancer was examined separately. For cottage cheese, all participants were divided into quartiles based on their respective daily consumption of cottage cheese, with the lowest group serving as the reference group. For low‐fat cream, low‐fat milk, skim milk, and yogurt, based on the distribution of daily consumption, non‐consumers served as the reference group, and the remaining participants were classified as tertiles of distribution.^23^

To better understand the dose–response association between low‐fat dairy consumption and the risk of developing lung cancer, a restricted cubic spline plot with three knots was employed to characterize lung cancer risk across the whole range of low‐fat dairy consumption. The regression coefficients of the second and third splines were considered to be equal to zero, and this null hypothesis was tested to get the *p*‐value for nonlinearity. To identify optimal intake of low‐fat dairy products for lung cancer prevention, the lowest HR for low‐fat dairy product consumption was also obtained.

To identify potential impact modifiers, a series of pre‐specified subgroup analyses were carried out after stratifying for age (≤65 vs. >65 years), gender (male vs. female), BMI (≤25 vs. >25 kg/m^2^), smoking status (never vs. current/former smokers), family history of lung cancer (no vs. yes/ possible), and daily caloric intake (≤median vs. >median). The significance of the interaction between the above stratification factors and low‐fat dairy consumption was examined using likelihood ratio tests. We also performed a series of sensitivity analyses to confirm the robustness of the results. The analysis was conducted again after the following participants were excluded: (i) those with missing data, in order to avoid the impact of missing data imputation on the results; (ii) those having a family history of lung cancer, because these participants were genetically more likely to develop lung cancer; (iii) lung cancer cases discovered within the first 2 or 4 years of follow‐up, with the purpose of removing possible reverse causality.

Statistical analyses were carried out using R software (version 4.2.1). *p* < 0.05 was regarded as statistically significant when a two‐tailed approach was used.

## 3 RESULTS

### 3.1 Baseline characteristics

In the current study, 98,459 subjects in total were included. The mean low‐fat dairy consumption was 133.96 g/day, with a standard deviation of 221.32 g/day. Individuals were divided into quartiles on the basis of their low‐fat dairy consumption levels as follows: Quartile 1, ≤6.50 g/day, *n* = 24,652; Quartile 2, >6.50 to ≤36.31 g/day, *n* = 24,579; Quartile 3, >36.31 to ≤161.08 g/day, *n* = 24,613; Quartile 4, >161.08 g/day, *n* = 24,615. Table 1 displays the baseline characteristics of the study population, split down by quartile. In comparison to individuals in the lowest quartile, those in the highest quartile were more likely to be female, white, never‐smokers, drinkers, had higher education levels, lower BMI, greater daily caloric and fiber intake, ate more fruits and vegetables, and consumed less red and processed meats.

**TABLE 1:** Baseline characteristics of study population according to low‐fat dairy consumption.

Characteristics	Overall	Quartiles of low‐fat dairy consumption (g/day)			
		Quartile 1 (≤6.50)	Quartile 2 (>6.50 to ≤36.31)	Quartile 3 (>36.31 to ≤161.08)	Quartile 4 (>161.08)
Number of participants	98,459	24,652	24,579	24,613	24,615
Low‐fat dairy consumption	133.96 ± 221.32	2.31 ± 2.03	16.99 ± 7.98	87.72 ± 36.37	428.85 ± 272.82
Age	65.52 ± 5.73	65.48 ± 5.78	65.51 ± 5.72	65.33 ± 5.64	65.76 ± 5.77
Gender					
Male	47,218 (47.96%)	14,781 (59.96%)	11,727 (47.71%)	10,342 (42.02%)	10,368 (42.12%)
Female	51,241 (52.04%)	9871 (40.04%)	12,852 (52.29%)	14,271 (57.98%)	14,247 (57.88%)
Race					
White	91,221 (92.65%)	21,439 (86.97%)	22,810 (92.80%)	23,203 (94.27%)	23,769 (96.56%)
Non‐white	7238 (7.35%)	3213 (13.03%)	1769 (7.20%)	1410 (5.73%)	846 (3.44%)
Education level					
College below	62,599 (63.58%)	17,568 (71.26%)	16,314 (66.37%)	14,865 (60.39%)	13,852 (56.27%)
College graduate/postgraduate	35,860 (36.42%)	7084 (28.74%)	8265 (33.63%)	9748 (39.61%)	10,763 (43.73%)
Marital status					
Married/living as married	77,374 (78.58%)	19,010 (77.11%)	19,424 (79.03%)	19,346 (78.60%)	19,594 (79.60%)
Others	21,085 (21.42%)	5642 (22.89%)	5155 (20.97%)	5267 (21.40%)	5021 (20.40%)
Body mass index (kg/m^2^)	27.20 ± 4.79	27.20 ± 4.69	27.53 ± 4.86	27.14 ± 4.85	26.94 ± 4.73
Smoking status					
Never	47,233 (47.97%)	10,332 (41.91%)	11,991 (48.79%)	12,057 (48.99%)	12,853 (52.22%)
Current/former	51,226 (52.03%)	14,320 (58.09%)	12,588 (51.21%)	12,556 (51.01%)	11,762 (47.78%)
Drinking status					
No	26,681 (27.10%)	7322 (29.70%)	6765 (27.52%)	6267 (25.46%)	6327 (25.70%)
Yes	71,778 (72.90%)	17,330 (70.30%)	17,814 (72.48%)	18,346 (74.54%)	18,288 (74.30%)
Trial arm					
Intervention	50,151 (50.94%)	12,469 (50.58%)	12,611 (51.31%)	12,685 (51.54%)	12,386 (50.32%)
Control	48,308 (49.06%)	12,183 (49.42%)	11,968 (48.69%)	11,928 (48.46%)	12,229 (49.68%)
Family history of lung cancer					
No	85,845 (87.19%)	21,210 (86.04%)	21,409 (87.10%)	21,562 (87.60%)	21,664 (88.01%)
Yes/possible	12,614 (12.81%)	3442 (13.96%)	3170 (12.90%)	3051 (12.40%)	2951 (11.99%)
Daily caloric intake (kcal)	1728.71 ± 658.04	1711.84 ± 692.11	1714.58 ± 663.23	1704.15 ± 648.62	1784.27 ± 623.12
Fruit (g/day)	275.24 ± 213.29	227.87 ± 214.94	259.32 ± 202.81	291.21 ± 208.36	322.60 ± 214.99
Vegetables (g/day)	284.83 ± 181.87	251.61 ± 174.69	281.62 ± 174.49	299.30 ± 181.82	306.85 ± 191.00
Fiber from diet (g/day)	18.03 ± 8.07	15.76 ± 7.54	17.23 ± 7.53	18.82 ± 7.91	20.29 ± 8.54
Red/processed meats (g/day)	12.26 ± 14.62	14.83 ± 16.84	13.15 ± 14.75	11.04 ± 13.29	10.01 ± 12.79

### 3.2 Association between intake of low‐fat dairy products and the risk of developing lung cancer

Over 869,807.9 person‐years of follow‐up, a total of 1642 cases of lung cancer were recorded. With an average follow‐up time of 8.84 years and a standard deviation of 1.94 years, the total incidence rate of lung cancer was 0.189 cases per 100 person‐years. Individuals in the highest quartile had a significantly reduced risk of lung cancer in contrast to the ones in the lowest quartile in the unadjusted model (HR_quartile 4 vs. 1_: 0.524, 95% CI: 0.456, 0.601, *p*_trend_ < 0.001) (Table 2). This inverse association persisted in the fully adjusted model (HR_quartile 4 vs. 1_: 0.769, 95% CI: 0.664, 0.891, *p*_trend_ = 0.005). The relationship between each individual component of low‐fat dairy products and the risk of developing lung cancer was also investigated. Lung cancer risk was found to have an inverse correlation for low‐fat cream (HR_quartiles 4 vs. 1_: 0.815, 95% CI: 0.668, 0.995, *p*_trend_ = 0.045), skim milk (HR_quartile 4 vs. 1_: 0.840, 95% CI: 0.715, 0.987, *p*_trend_ = 0.035), and yogurt (HR_quartile 4 vs. 1_: 0.682, 95% CI: 0.575, 0.808, *p*_trend_ < 0.001). However, no significant association between consuming cottage cheese and low‐fat milk and the risk of lung cancer was discovered (*p*_trend_ > 0.05).

**TABLE 2:** Hazard ratios of the association of low‐fat dairy consumption with lung cancer risk.

Quartiles of low‐fat dairy consumption (g/day)	Number of cases	Incidence rate per 100 person‐years (95% CI)	Hazard ratio (95% CI)		
			Unadjusted	Model 1^a^	Model 2^b^
Overall					
Quartile 1 (≤6.50)	574	0.268 (0.247, 0.291)	1.000 (reference)	1.000 (reference)	1.000 (reference)
Quartile 2 (>6.50 to ≤36.31)	416	0.192 (0.175, 0.212)	0.716 (0.631, 0.812)	0.736 (0.648, 0.836)	0.874 (0.769, 0.994)
Quartile 3 (>36.31 to ≤161.08)	341	0.156 (0.140, 0.173)	0.580 (0.508, 0.664)	0.614 (0.536, 0.703)	0.796 (0.692, 0.915)
Quartile 4 (>161.08)	311	0.141 (0.126, 0.157)	0.524 (0.456, 0.601)	0.536 (0.466, 0.617)	0.769 (0.664, 0.891)
*p*_trend_			<0.001	<0.001	0.005
Cottage cheese					
Quartile 1 (≤0.29)	456	0.209 (0.191, 0.229)	1.000 (reference)	1.000 (reference)	1.000 (reference)
Quartile 2 (>0.29 to ≤3.51)	476	0.178 (0.163, 0.195)	0.850 (0.748, 0.967)	0.843 (0.740, 0.960)	0.875 (0.768, 0.997)
Quartile 3 (>3.51 to ≤9.31)	361	0.174 (0.157, 0.193)	0.831 (0.724, 0.954)	0.806 (0.700, 0.928)	0.859 (0.746, 0.990)
Quartile 4 (>9.31)	349	0.196 (0.177, 0.218)	0.937 (0.815, 1.077)	0.827 (0.717, 0.954)	0.917 (0.793, 1.060)
*p*_trend_			0.767	0.131	0.808
Low‐fat cream^c^					
Quartile 1 (0)	1357	0.215 (0.204, 0.227)	1.000 (reference)	1.000 (reference)	1.000 (reference)
Quartile 2 (>0 to ≤0.12)	91	0.111 (0.090, 0.136)	0.516 (0.417, 0.638)	0.574 (0.464, 0.711)	0.702 (0.567, 0.870)
Quartile 3 (>0.12 to ≤0.55)	86	0.112 (0.091, 0.138)	0.519 (0.417, 0.645)	0.592 (0.475, 0.737)	0.715 (0.573, 0.891)
Quartile 4 (>0.55)	108	0.136 (0.112, 0.164)	0.628 (0.516, 0.764)	0.701 (0.576, 0.854)	0.815 (0.668, 0.995)
*p*_trend_			<0.001	<0.001	0.045
Low‐fat milk^c^					
Quartile 1 (0)	916	0.222 (0.208, 0.237)	1.000 (reference)	1.000 (reference)	1.000 (reference)
Quartile 2 (>0 to ≤2.22)	259	0.162 (0.143, 0.183)	0.725 (0.632, 0.833)	0.776 (0.675, 0.891)	0.923 (0.800, 1.064)
Quartile 3 (>2.22 to ≤8.80)	228	0.150 (0.131, 0.170)	0.671 (0.580, 0.776)	0.701 (0.606, 0.810)	0.922 (0.795, 1.068)
Quartile 4 (>8.80)	239	0.164 (0.145, 0.186)	0.736 (0.639, 0.849)	0.695 (0.602, 0.801)	0.894 (0.773, 1.034)
*p*_trend_			<0.001	<0.001	0.171
Skim milk^c^					
Quartile 1 (0)	1099	0.219 (0.206, 0.232)	1.000 (reference)	1.000 (reference)	1.000 (reference)
Quartile 2 (>0 to ≤79.10)	189	0.154 (0.134, 0.178)	0.704 (0.603, 0.821)	0.764 (0.654, 0.892)	0.866 (0.740, 1.014)
Quartile 3 (>79.10 to ≤267.34)	176	0.142 (0.123, 0.165)	0.648 (0.552, 0.759)	0.668 (0.569, 0.783)	0.848 (0.720, 0.998)
Quartile 4 (>267.34)	178	0.146 (0.126, 0.169)	0.666 (0.569, 0.781)	0.650 (0.555, 0.762)	0.840 (0.715, 0.987)
*p*_trend_			<0.001	<0.001	0.035
Yogurt ^c^					
Quartile 1 (0)	944	0.276 (0.259, 0.294)	1.000 (reference)	1.000 (reference)	1.000 (reference)
Quartile 2 (>0 to ≤5.37)	324	0.160 (0.143, 0.178)	0.578 (0.509, 0.656)	0.646 (0.568, 0.735)	0.792 (0.695, 0.903)
Quartile 3 (>5.37 to ≤14.39)	192	0.117 (0.101, 0.135)	0.423 (0.362, 0.494)	0.469 (0.401, 0.549)	0.648 (0.553, 0.761)
Quartile 4 (>14.39)	182	0.114 (0.098, 0.131)	0.411 (0.351, 0.482)	0.484 (0.412, 0.570)	0.682 (0.575, 0.808)
*p*_trend_			<0.001	<0.001	<0.001

^a^ Model 1: model 1 was controlled with age, gender, and race/ethnic.

^b^ Model 2: model 2 was additionally controlled with marital status, educational level, body mass index, smoking status, drinking status, trial arm, family history of lung cancer, daily caloric intake (kcal), fruit consumption (g/day), vegetable consumption (g/day), red and processed meat consumption (g/day), and dietary fiber from diet (g/day).

^c^ For low‐fat cream, low‐fat milk, skim milk, and yogurt, non‐consumers served as the reference group, and the remaining participants were classified as tertiles of distribution.

### 3.3 Additional analyses

**FIGURE 2:**
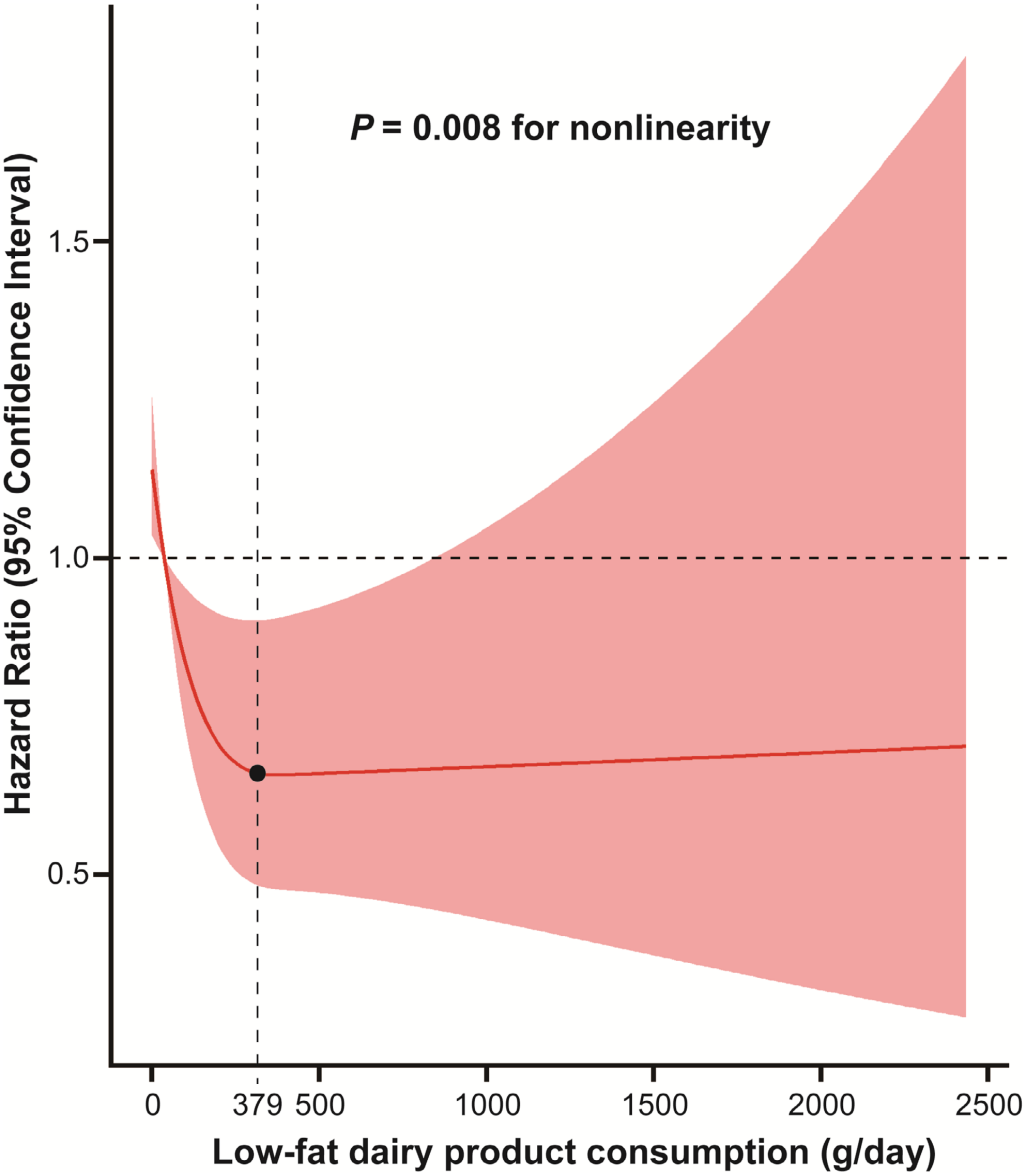
Dose–response association between low‐fat dairy consumption and the risk of lung cancer.

A restricted cubic spline plot was utilized to determine the link between the intake of low‐fat dairy products and the risk of developing lung cancer across the full range of consumption levels. The outcomes of the analysis showed a nonlinear dose–response relationship between low‐fat dairy consumption and the risk of developing lung cancer (*p*_nonlinearity_ = 0.008) (Figure 2). Specifically, within a certain range, lung cancer risk was significantly reduced with the high consumption of low‐fat dairy products. However, this decreasing trend leveled off when low‐fat dairy consumption exceeded 379 g/day. No significant interactions were observed between lung cancer risk and factors such as age, gender, BMI, smoking status, and family history of lung cancer in the subgroup analysis (all *p*_interaction_ >0.05) (Table 3). However, for participants with higher daily caloric intake, greater intake of low‐fat dairy products may have a stronger protective effect against lung cancer (HR_quartile 4 vs. 1_: 0.766, 95% CI: 0.612, 0.958, *p*_interaction_ = 0.031). When participants with missing data, a family history of lung cancer, and lung cancer cases discovered within the first 2 or 4 years of follow‐up were excluded from the study, the findings were still robust in sensitivity analyses (Table 4).

**TABLE 3:** Subgroup analyses on the association of low‐fat dairy consumption with the risk of lung cancer.

Subgroup variable	Number of participants	Number of cases	HR_Quartile 4 vs. Quartile 1_ (95% CI)^a^	*p*_interaction_
Age (years)				0.748
≤65	25,291	308	0.929 (0.709, 1.217)	
>65	23,976	577	0.904 (0.747, 1.093)	
Gender				0.847
Male	25,149	556	0.942 (0.770, 1.151)	
Female	24,118	329	0.854 (0.667, 1.093)	
Body mass index (kg/m^2^)				0.292
≤25	17,182	357	0.748 (0.580, 0.964)	
>25	32,085	528	1.034 (0.850, 1.258)	
Smoking status				0.109
Never	23,185	63	0.909 (0.528, 1.564)	
Current/former	26,082	822	0.775 (0.660, 0.910)	
Family history of lung cancer				0.341
No	42,874	705	0.868 (0.730, 1.033)	
Yes/possible	6393	180	1.142 (0.806, 1.617)	
Daily caloric intake (kcal)				0.031
≤Medium	24,635	461	1.073 (0.866, 1.330)	
>Medium	24,632	424	0.766 (0.612, 0.958)	

Abbreviations: BMI, body mass index; CI, confidence interval; HR, hazard ratio.

^a^ HRs were controlled with age, gender, race/ethnic, marital status, educational level, BMI, smoking status, drinking status, trial arm, family history of lung cancer, daily caloric intake (kcal), fruit consumption (g/day), vegetable consumption (g/day), red and processed meat consumption (g/day), and fiber from diet (g/day).

**TABLE 4:** Sensitivity analyses on the association of low‐fat dairy consumption with the risk of lung cancer.

Categories	HR_Quartile 4 vs. Quartile 1_ (95% CI)^a^	*p*_trend_
Excluded participants with missing data	0.773 (0.666, 0.896)	0.005
Excluded participants with family history of lung cancer^b^	0.728 (0.617, 0.858)	0.004
Excluded cases observed within the first 2 years	0.760 (0.646, 0.895)	0.006
Excluded cases observed within the first 4 years	0.718 (0.595, 0.866)	0.005

Abbreviations: CI, confidence interval; HR, hazard ratio.

^a^ HRs were adjusted for age (years), sex (male, female), race/ethnic (white, non‐white), marital status (married or living as married, others), educational level (college below, college graduate or postgraduate), body mass index (kg/m^2^), smoking status (never smoker, current or former smoker), drinking status (no, yes), trial arm (intervention, control), family history of lung cancer (no, yes or possible), daily caloric intake (kcal), fruit consumption (g/day), vegetable consumption (g/day), red and processed meat consumption (g/day), and dietary fiber from diet (g/day).

^b^ HR was not adjusted for family history of lung cancer.

## 4 DISCUSSION

According to the results of the prospective PLCO trial, the current investigation demonstrated a link between consuming more low‐fat dairy products and a decreased risk of developing lung cancer. After taking into account potential confounders, the inverse relationship persisted. A nonlinear dose–response relationship between low‐fat dairy consumption and the risk of developing lung cancer was suggested by the restricted cubic spline plot. Subgroup analyses showed that increased low‐fat dairy consumption might have greater benefits for lung cancer prevention in individuals with higher daily caloric intake. Additionally, the results were robust in sensitivity analyses, as they remained consistent after excluding participants who may have influenced the results.

Numerous reports have recently focused on the link between dairy product intake and different types of cancer. Higher dairy milk consumption was linked to an increased risk of breast cancer, according to a cohort study of 52,795 North American women who were followed up for 7.9 years.^8^ Moreover, a prospective study of 28,737 participants demonstrated a 27% increased risk of prostate carcinoma in males with high dairy consumption in contrast to the ones with low dairy consumption.^24^ According to a meta‐analysis, there is a consistent inverse correlation between dairy consumption and the risk of developing prostate cancer.^25^ In contrast, according to a prospective observational study, increased dairy consumption significantly reduced the risk of developing colorectal cancer by up to 45% among older Mediterranean individuals.^26^ The positive effect of dairy products on colorectal carcinoma prevention has also been demonstrated elsewhere.^10^, ^27^ Furthermore, a pooled analysis published in 2020 showed that participants with high yogurt consumption had a 19% lower lung cancer risk compared to no yogurt consumers.^9^ However, no link was found between milk, dairy consumption, and the risk of developing lung cancer.^10^, ^28^ These contradictory results could create confusion about whether dairy product consumption should be increased or decreased to minimize cancer risk. These inconsistencies may be attributed to the fact that dairy products are a complex and diverse food group, and previous researchers have failed to consider the effect of different fat contents and types of dairy products on the outcomes.

To our knowledge, there has not been any published study that has investigated the link between consuming low‐fat dairy and lung cancer risk. In this study, it was observed that individuals with the highest intake of low‐fat dairy had approximately a 48% reduction in lung cancer risk compared to those with the least consumption. Increased use of low‐fat dairy products still decreased the risk of lung cancer by roughly 23%, even after adjusting for potential confounding factors. The restricted cubic spline plot revealed a nonlinear dose–response relationship between low‐fat dairy consumption and the risk of developing lung cancer, indicating the greatest risk reduction at relatively low doses of intake. No further risk reduction was observed for low‐fat dairy intake greater than 379 g/day, suggesting that although low‐fat dairy products are beneficial for lung cancer prevention, it is not advisable to excessively increase their intake. In the analysis of the individual components, it was found that a greater intake of low‐fat cream, skim milk, and yogurt was linked to a lower risk of developing lung cancer. Nevertheless, no link between the consumption of cottage cheese and low‐fat milk and the risk of lung cancer was discovered. This suggests that low‐fat cream, skim milk, and yogurt may serve as main contributors to the inverse correlation between low‐fat dairy consumption and lung cancer risk, encouraging individuals to consume more of these three dairy products. Overall, the results indicate the possibility that an appropriate increase in the use of various low‐fat dairy products may help prevent lung cancer.

The observed inverse relationship in this study can be elucidated through several possible mechanisms, which are as follows: (i) dairy products with high saturated fatty acids have been linked to heightened levels of inflammation and oxidative stress, both of which intersect with the pathogenesis of lung cancer.^29^, ^30^ However, by removing a substantial portion of saturated fats, low‐fat dairy products effectively diminish this unfavorable effect. (ii) Low‐fat dairy products are bestowed with an abundant supply of vital nutrients such as calcium, vitamin D, conjugated linoleic acids, whey protein and casein.^13^ Mounting evidence indicates that these components manifest robust anticancer properties through their involvement in diverse pathways, encompassing the activation of apoptosis, modulation of autophagy, attenuation of inflammation, and modulation of immune responses.^31^, ^32^ (iii) Fermented dairy products, such as yogurt and cheese, are valuable sources of probiotics. On the one hand, probiotics, such as *Bifidobacterium bifidum* and *lactobacillus*, can directly exert anti‐tumor effects by activating immune signaling molecules and immune cells, anti‐proliferative activity, induction of apoptosis, cell cycle arrest, and exertion of anti‐angiogenic.^33^, ^34^, ^35^ Furthermore, animal experiments have uncovered a compelling link between the composition of gut microbiota and lung microbiota,^36^ with the former being susceptible to dietary influences. Notably, probiotics have garnered recognition for their ability to modulate the composition of intestinal flora.^37^ Consequently, it is conceivable that they may exert a favorable influence on the immune milieu within the lungs, thereby reducing the risk of lung cancer. (iv) Previous evidence has demonstrated that insulin resistance constitutes a significant risk factor for lung cancer.^38^ The consumption of low‐fat dairy products has been associated with improvements in insulin resistance,^39^ providing a potential explanation for the protective effect of low‐fat dairy against the development of lung cancer. Nonetheless, further research endeavors are warranted to corroborate these underlying mechanisms and shed more light on their precise contributions.

It was discovered that participants with higher daily caloric intake exhibited a prominent robust preventive effect of low‐fat dairy products against lung cancer, suggesting a greater need to increase low‐fat dairy consumption in people with a high‐calorie diet for lung cancer prevention. We posit that this observation can be attributed to the following factors: (i) people who adherence to a high‐calorie diet usually consume more fried, baked, and high‐fat foods in their diet, which may induce nutritional imbalances, such as deficiencies in essential vitamins and minerals. Insufficient or unbalanced nutrient intake can weaken the immune system, impair cellular function, and increase oxidative stress,^40^, ^41^ thus potentially contributing to the development of lung cancer. Low‐fat dairy products, abundant in nutrients like protein, vitamins, and calcium, serve as valuable sources to address potential nutrient deficiencies stemming from high‐calorie diets. By providing essential supplements, they contribute to reducing the risk of lung cancer. (ii) The high‐calorie diet induces a state of subclinical tissue inflammation, leading to insulin resistance.^42^ This implies that individuals following a high‐calorie diet often experience heightened insulin resistance. For such individuals, the consumption of low‐fat dairy products offers greater advantages in mitigating insulin resistance.^39^ (iii) Previous evidence indicates that a high‐calorie diet disrupts the microbial community balance in the body, which can have detrimental effects on the immune environment in the lungs.^36^, ^43^ In individuals following high‐calorie diets, the consumption of low‐fat dairy products can partially counteract this adverse effect. This is due to the rich probiotic content of low‐fat dairy products, which has a favorable impact on regulating the intestinal flora.^37^ Further research is necessary to validate these hypotheses.

This study has clear advantages. It was demonstrated for the first time that greater low‐fat dairy consumption is linked to a reduced risk of lung cancer. This outcome, as well as the optimal daily consumption levels of low‐fat dairy products that were calculated in the dose–response relationship analysis, could help update the dietary guidance on lung cancer prevention, especially for people who adherence to high‐calorie diets. Moreover, in the analysis of the individual low‐fat dairy components, types of low‐fat dairy products (low‐fat cream, skim milk, and yogurt) were identified, which are potentially more effective in lung cancer prevention. This could be used as a reference for recommending the variety and daily consumption amounts of low‐fat dairy products. The prospective study design using a large population as well as robust results affirm the credibility of our study. Moreover, the sufficiently long follow‐up period ensures that the outcome events can be observed within the time frame.

However, several limitations of this study must be acknowledged. The study population consisted mainly of older adults in the United States. Therefore, we have reservations about whether low‐fat dairy products can also help prevent lung cancer for young people in the United States. For the same reason, the results of this research may not apply to other regions and populations. Moreover, Dietary history information was self‐reported by the participants; while this might have introduced non‐differential bias, it is often unavoidable in epidemiological surveys. Nonetheless, the DHQ is an excellent dietary assessment tool, and its validity has been well established.^16^ Therefore, the effect of the non‐differential bias might not be significant. Furthermore, the absence of a standard method to categorize low‐fat dairy product consumption levels leads to base the analysis on the categorization of quartiles within the study population. Finally, since the dietary history information was collected only once, the current study does not consider changes in the dietary habits of individuals over the follow‐up time. However, the dietary habits of individuals do not change dramatically and studies using a single measurement of dietary information often yield weaker association indicators.^44^ This study, despite using one‐time measure, provides a strong association, suggesting that low‐fat dairy products have a definitive preventive impact against lung cancer and are even more significant than the results presented in this study.

## 5 CONCLUSION

According to this study, consuming more low‐fat dairy products is significantly linked to a lower risk of developing lung cancer in the US population, indicating that a suitable increase in the use of low‐fat dairy products may have a preventive impact against lung cancer. However, additional epidemiological research is required to confirm these findings.

## AUTHOR CONTRIBUTIONS

**Zhiyong Zhu:** Data curation (lead); formal analysis (equal); writing – original draft (lead). **Linglong Peng:** Conceptualization (equal); data curation (equal); software (lead). **He Zhou:** Methodology (equal); software (supporting). **Haitao Gu:** Funding acquisition (supporting); investigation (equal). **Yunhao Tang:** Project administration (supporting); visualization (supporting). **Zhihang Zhou:** Software (supporting). **Yaxu Wang:** Funding acquisition (lead); supervision (equal); writing – review and editing (equal). **Ling Xiang:** Supervision (lead); validation (equal); writing – review and editing (supporting).

## FUNDING STATEMENT

This work was funded by the General Project of Chongqing Natural Science Foundation, Chongqing Science and Technology Commission, China (cstc2021jcyj‐msxmX0112, CSTB2022NSCQ‐MSX1005, and cstc2021jcyj‐msxmX0153).

## CONFLICT OF INTEREST STATEMENT

No competing interests exist.

## ETHICS STATEMENT

The PLCO trial was approved by the Institutional Review Board of the National Cancer Institute and was carried out following the rules of the Declaration of Helsinki. All participants gave their informed consent for inclusion before they participated in this trial.

## Supporting information


Table S1.

## Data Availability

All the raw data used in this study were collected from the PLCO trial and can be obtained with the consent of the NCI (https://cdas.cancer.gov/plco/).

## References

[1] Sung H , Ferlay J , Siegel RL , et al. Global cancer statistics 2020: GLOBOCAN estimates of incidence and mortality worldwide for 36 cancers in 185 countries. CA Cancer J Clin. 2021;71(3):209‐249. 33538338 10.3322/caac.21660

[2] Kim JW , Marquez CP , Kostyrko K , et al. Antitumor activity of an engineered decoy receptor targeting CLCF1‐CNTFR signaling in lung adenocarcinoma. Nat Med. 2019;25(11):1783‐1795. 31700175 10.1038/s41591-019-0612-2 PMC7087454

[3] Abbosh C , Birkbak NJ , Wilson GA , et al. Phylogenetic ctDNA analysis depicts early‐stage lung cancer evolution. Nature. 2017;545(7655):446‐451. 28445469 10.1038/nature22364 PMC5812436

[4] Cai H , Sobue T , Kitamura T , et al. Dietary fibre intake is associated with reduced risk of lung cancer: a Japan public health centre‐based prospective study (JPHC). Int J Epidemiol. 2022;51(4):1142‐1152. 35353155 10.1093/ije/dyac054

[5] Vieira AR , Abar L , Vingeliene S , et al. Fruits, vegetables and lung cancer risk: a systematic review and meta‐analysis. Ann Oncol. 2016;27(1):81‐96. 26371287 10.1093/annonc/mdv381

[6] Lam TK , Cross AJ , Consonni D , et al. Intakes of red meat, processed meat, and meat mutagens increase lung cancer risk. Cancer Res. 2009;69(3):932‐939. 19141639 10.1158/0008-5472.CAN-08-3162 PMC2720759

[7] López‐Plaza B , Bermejo LM , Santurino C , Cavero‐Redondo I , Álvarez‐Bueno C , Gómez‐Candela C . Milk and dairy product consumption and prostate cancer risk and mortality: an overview of systematic reviews and meta‐analyses. Adv Nutr. 2019;10(suppl_2):S212‐s23. 31089741 10.1093/advances/nmz014 PMC6518142

[8] Fraser GE , Jaceldo‐Siegl K , Orlich M , Mashchak A , Sirirat R , Knutsen S . Dairy, soy, and risk of breast cancer: those confounded milks. Int J Epidemiol. 2020;49(5):1526‐1537. 32095830 10.1093/ije/dyaa007 PMC8453418

[9] Yang JJ , Yu D , Xiang YB , et al. Association of dietary fiber and yogurt consumption with lung cancer risk: a pooled analysis. JAMA Oncol. 2020;6(2):e194107. 31647500 10.1001/jamaoncol.2019.4107 PMC6813596

[10] Lumsden AL , Mulugeta A , Hyppönen E . Milk consumption and risk of twelve cancers: a large‐scale observational and Mendelian randomisation study. Clin Nutr. 2023;42(1):1‐8. 36473423 10.1016/j.clnu.2022.11.006

[11] Park SY , Murphy SP , Wilkens LR , Stram DO , Henderson BE , Kolonel LN . Calcium, vitamin D, and dairy product intake and prostate cancer risk: the multiethnic cohort study. Am J Epidemiol. 2007;166(11):1259‐1269. 17925283 10.1093/aje/kwm269

[12] Jansen RJ , Robinson DP , Frank RD , et al. Fatty acids found in dairy, protein and unsaturated fatty acids are associated with risk of pancreatic cancer in a case‐control study. Int J Cancer. 2014;134(8):1935‐1946. 24590454 10.1002/ijc.28525 PMC3942799

[13] Aguilera‐Buenosvinos I , Fernandez‐Lazaro CI , Romanos‐Nanclares A , et al. Dairy consumption and incidence of breast cancer in the 'Seguimiento Universidad de Navarra' (SUN) project. Nutrients. 2021;13(2):687. 33669972 10.3390/nu13020687 PMC7924827

[14] Yakoob MY , Shi P , Willett WC , et al. Circulating biomarkers of dairy fat and risk of incident diabetes mellitus among men and women in the United States in two large prospective cohorts. Circulation. 2016;133(17):1645‐1654. 27006479 10.1161/CIRCULATIONAHA.115.018410 PMC4928633

[15] Buys SS , Partridge E , Black A , et al. Effect of screening on ovarian cancer mortality: the prostate, lung, colorectal and ovarian (PLCO) cancer screening randomized controlled trial. JAMA. 2011;305(22):2295‐2303. 21642681 10.1001/jama.2011.766

[16] Subar AF , Thompson FE , Kipnis V , et al. Comparative validation of the Block, Willett, and National Cancer Institute food frequency questionnaires: the Eating at America's Table Study. Am J Epidemiol. 2001;154(12):1089‐1099. 11744511 10.1093/aje/154.12.1089

[17] Huang Y , Liu F , Chen AM , et al. Type 2 diabetes prevention diet and the risk of pancreatic cancer: a large prospective multicenter study. Clin Nutr. 2021;40(11):5595‐5604. 34656956 10.1016/j.clnu.2021.09.037

[18] Satija A , Bhupathiraju SN , Spiegelman D , et al. Healthful and unhealthful plant‐based diets and the risk of coronary heart disease in U.S. adults. J Am Coll Cardiol. 2017;70(4):411‐422. 28728684 10.1016/j.jacc.2017.05.047 PMC5555375

[19] Hughes KC , Gao X , Kim IY , et al. Intake of dairy foods and risk of Parkinson disease. Neurology. 2017;89(1):46‐52. 28596209 10.1212/WNL.0000000000004057 PMC5496517

[20] Pettersson A , Kasperzyk JL , Kenfield SA , et al. Milk and dairy consumption among men with prostate cancer and risk of metastases and prostate cancer death. Cancer Epidemiol Biomarkers Prev. 2012;21(3):428‐436. 22315365 10.1158/1055-9965.EPI-11-1004 PMC3297731

[21] Srour B , Fezeu LK , Kesse‐Guyot E , et al. Ultra‐processed food intake and risk of cardiovascular disease: prospective cohort study (NutriNet‐Santé). BMJ. 2019;365:l1451. 31142457 10.1136/bmj.l1451 PMC6538975

[22] Zhang Y , Zhong G , Zhu M , Chen L , Wan H , Luo F . Association between diabetes risk reduction diet and lung cancer risk in 98,159 participants: results from a prospective study. Front Oncol. 2022;12:855101. 35574372 10.3389/fonc.2022.855101 PMC9097267

[23] Wang T , Zhu Y , Zheng Y , et al. Dairy consumption and risk of esophagus cancer in the prostate, lung, colorectal, and ovarian cohort. Front Nutr. 2022;9:1015062. 36570164 10.3389/fnut.2022.1015062 PMC9773090

[24] Orlich MJ , Mashchak AD , Jaceldo‐Siegl K , et al. Dairy foods, calcium intakes, and risk of incident prostate cancer in Adventist Health Study‐2. Am J Clin Nutr. 2022;116(2):314‐324. 35672028 10.1093/ajcn/nqac093 PMC9348981

[25] Aune D , Navarro Rosenblatt DA , Chan DS , et al. Dairy products, calcium, and prostate cancer risk: a systematic review and meta‐analysis of cohort studies. Am J Clin Nutr. 2015;101(1):87‐117. 25527754 10.3945/ajcn.113.067157

[26] Barrubés L , Babio N , Mena‐Sánchez G , et al. Dairy product consumption and risk of colorectal cancer in an older mediterranean population at high cardiovascular risk. Int J Cancer. 2018;143(6):1356‐1366. 29663376 10.1002/ijc.31540

[27] Papadimitriou N , Markozannes G , Kanellopoulou A , et al. An umbrella review of the evidence associating diet and cancer risk at 11 anatomical sites. Nat Commun. 2021;12(1):4579. 34321471 10.1038/s41467-021-24861-8 PMC8319326

[28] Yu Y , Li H , Xu K , et al. Dairy consumption and lung cancer risk: a meta‐analysis of prospective cohort studies. Onco Targets Ther. 2016;9:111‐116. 26766916 10.2147/OTT.S95714 PMC4699511

[29] Tucker LA . Milk fat intake and telomere length in U.S. women and men: the role of the Milk fat fraction. Oxid Med Cell Longev. 2019;2019:1574021. 31772698 10.1155/2019/1574021 PMC6855010

[30] Jin C , Lagoudas GK , Zhao C , et al. Commensal microbiota promote lung cancer development via γδ T cells. Cell. 2019;176(5):998‐1013.e16. 30712876 10.1016/j.cell.2018.12.040 PMC6691977

[31] Tavera‐Mendoza LE , Westerling T , Libby E , et al. Vitamin D receptor regulates autophagy in the normal mammary gland and in luminal breast cancer cells. Proc Natl Acad Sci USA. 2017;114(11):E2186‐E2194. 28242709 10.1073/pnas.1615015114 PMC5358377

[32] Bocca C , Bozzo F , Cannito S , Colombatto S , Miglietta A . CLA reduces breast cancer cell growth and invasion through ERalpha and PI3K/Akt pathways. Chem Biol Interact. 2010;183(1):187‐193. 19800873 10.1016/j.cbi.2009.09.022

[33] Shi Y , Zheng W , Yang K , et al. Intratumoral accumulation of gut microbiota facilitates CD47‐based immunotherapy via STING signaling. J Exp Med. 2020;217(5):e20192282. 32142585 10.1084/jem.20192282 PMC7201921

[34] Dasari S , Kathera C , Janardhan A , Praveen Kumar A , Viswanath B . Surfacing role of probiotics in cancer prophylaxis and therapy: a systematic review. Clin Nutr. 2017;36(6):1465‐1472. 27923508 10.1016/j.clnu.2016.11.017

[35] Foster KR , Schluter J , Coyte KZ , Rakoff‐Nahoum S . The evolution of the host microbiome as an ecosystem on a leash. Nature. 2017;548(7665):43‐51. 28770836 10.1038/nature23292 PMC5749636

[36] Trompette A , Gollwitzer ES , Yadava K , et al. Gut microbiota metabolism of dietary fiber influences allergic airway disease and hematopoiesis. Nat Med. 2014;20(2):159‐166. 24390308 10.1038/nm.3444

[37] Kitada Y , Muramatsu K , Toju H , et al. Bioactive polyamine production by a novel hybrid system comprising multiple indigenous gut bacterial strategies. Sci Adv. 2018;4(6):eaat0062. 29963630 10.1126/sciadv.aat0062 PMC6021145

[38] Argirion I , Weinstein SJ , Männistö S , Albanes D , Mondul AM . Serum insulin, glucose, indices of insulin resistance, and risk of lung cancer. Cancer Epidemiol Biomarkers Prevent. 2017;26(10):1519‐1524. 10.1158/1055-9965.EPI-17-0293 PMC5626607 28698186

[39] Rideout TC , Marinangeli CP , Martin H , Browne RW , Rempel CB . Consumption of low‐fat dairy foods for 6 months improves insulin resistance without adversely affecting lipids or bodyweight in healthy adults: a randomized free‐living cross‐over study. Nutr J. 2013;12:56. 23638799 10.1186/1475-2891-12-56 PMC3651862

[40] Kishton RJ , Sukumar M , Restifo NP . Metabolic regulation of T cell longevity and function in tumor immunotherapy. Cell Metab. 2017;26(1):94‐109. 28683298 10.1016/j.cmet.2017.06.016 PMC5543711

[41] Wu D , Lewis ED , Pae M , Meydani SN . Nutritional modulation of immune function: analysis of evidence, mechanisms, and clinical relevance. Front Immunol. 2018;9:3160. 30697214 10.3389/fimmu.2018.03160 PMC6340979

[42] Perakakis N , Triantafyllou GA , Fernández‐Real JM , et al. Physiology and role of irisin in glucose homeostasis. Nat Rev Endocrinol. 2017;13(6):324‐337. 28211512 10.1038/nrendo.2016.221 PMC5878942

[43] Jin H , Zhang C . High fat high calories diet (HFD) increase gut susceptibility to carcinogens by altering the gut microbial community. J Cancer. 2020;11(14):4091‐4098. 32368291 10.7150/jca.43561 PMC7196248

[44] Zhang J , Zhao A , Wu W , et al. Beneficial effect of dietary diversity on the risk of disability in activities of daily living in adults: a prospective cohort study. Nutrients. 2020;12(11):3263. 33113764 10.3390/nu12113263 PMC7692387

