# Supplementary material for: Low‐fat dairy consumption and the risk of lung cancer: A large prospective cohort study
Source: Cancer Med. 2023 Jun 16;12(15):16558–69. doi: 10.1002/cam4.6249 (PMC10469841; doi:10.1002/cam4.6249)
Supplement: Supplementary file 1 — Table S1. [file CAM4-12-16558-s001.docx]

**Supplemental Table 1.** Distribution of covariates with missing data before and after imputation^*^

| Variable | Before imputation | After imputation | Number (%) with missing data |
| --- | --- | --- | --- |
| ***Family history of lung cancer*** |  |  | 757 (0.77%) |
| No | 85088 (87.09%) | 85845 (87.19%) |  |
| Yes | 10266 (10.51%) | 10266 (10.43%) |  |
| Possible | 2348 (2.40%) | 2348 (2.38%) |  |
| ***Race*** |  |  | 34 (0.03%) |
| White | 91187(92.65%) | 91221(92.65%) |  |
| Non-White | 7238(7.35%) | 7238(7.35%) |  |
| ***Smoking status*** |  |  | 20 (0.02%) |
| Never | 47213 (47.96%) | 47233 (47.97%) |  |
| Current | 8993 (9.14%) | 8993 (9.14%) |  |
| Former | 42233 (42.90%) | 42233 (42.89%) |  |
| ***Marital status*** |  |  | 185 (0.19%) |
| Married/living as married | 77189(78.54%) | 77374(78.58%) |  |
| Others | 21085(21.46%) | 21085(21.42%) |  |
| ***Education level*** |  |  | 196 (0.20%) |
| College below | 62403(63.51%) | 62599(63.58%) |  |
| College graduate | 17353(17.66%) | 17353(17.62%) |  |
| Postgraduate | 18507(18.83%) | 18507(18.80%) |  |
| ***Body mass index (kg/m^2^)*** | 27.21±4.82 | 27.20±4.79 | 1293 (1.31%) |
